# Supplementary material for: Multiparticulate Systems of Meloxicam for Colonic Administration in Cancer or Autoimmune Diseases
Source: Pharmaceutics. 2022 Jul 20;14(7):1504. doi: 10.3390/pharmaceutics14071504 (PMC9322124; doi:10.3390/pharmaceutics14071504)
Supplement: Supplementary file 1 [file pharmaceutics-14-01504-s001.zip › pharmaceutics-1753133-supplementary.pdf]

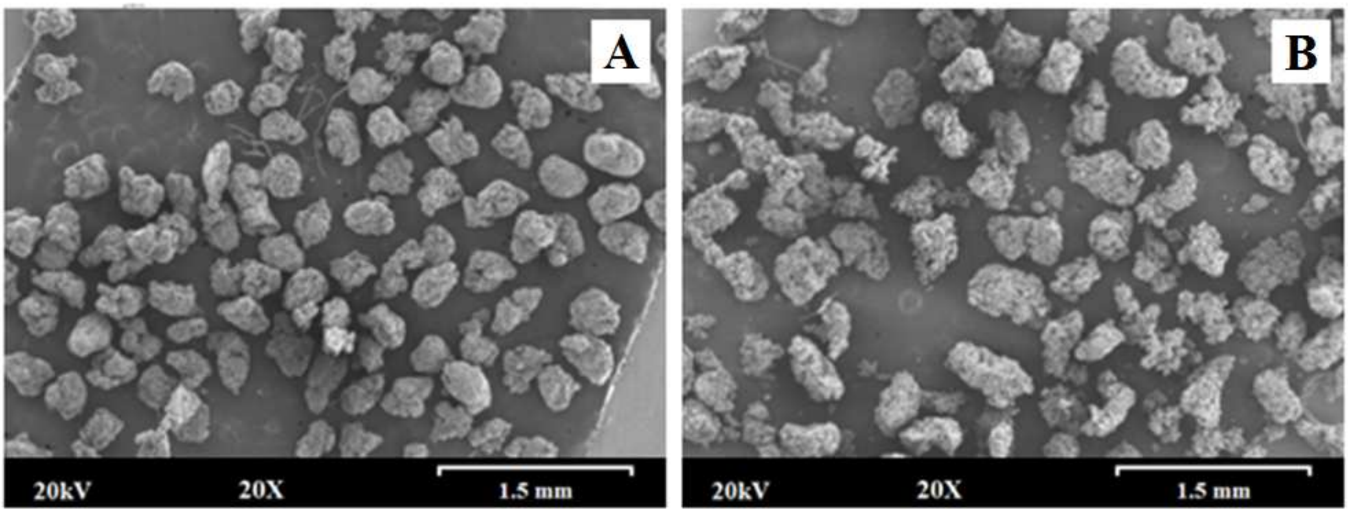

Figure S1. Scanning electron micrographs of CNM multiparticulate system and its blank formulation, at a magnification of 20× and the scale bar is equal to 1.5 mm: (A) CNM multiparticulate system; (B) blank CNM formulation.

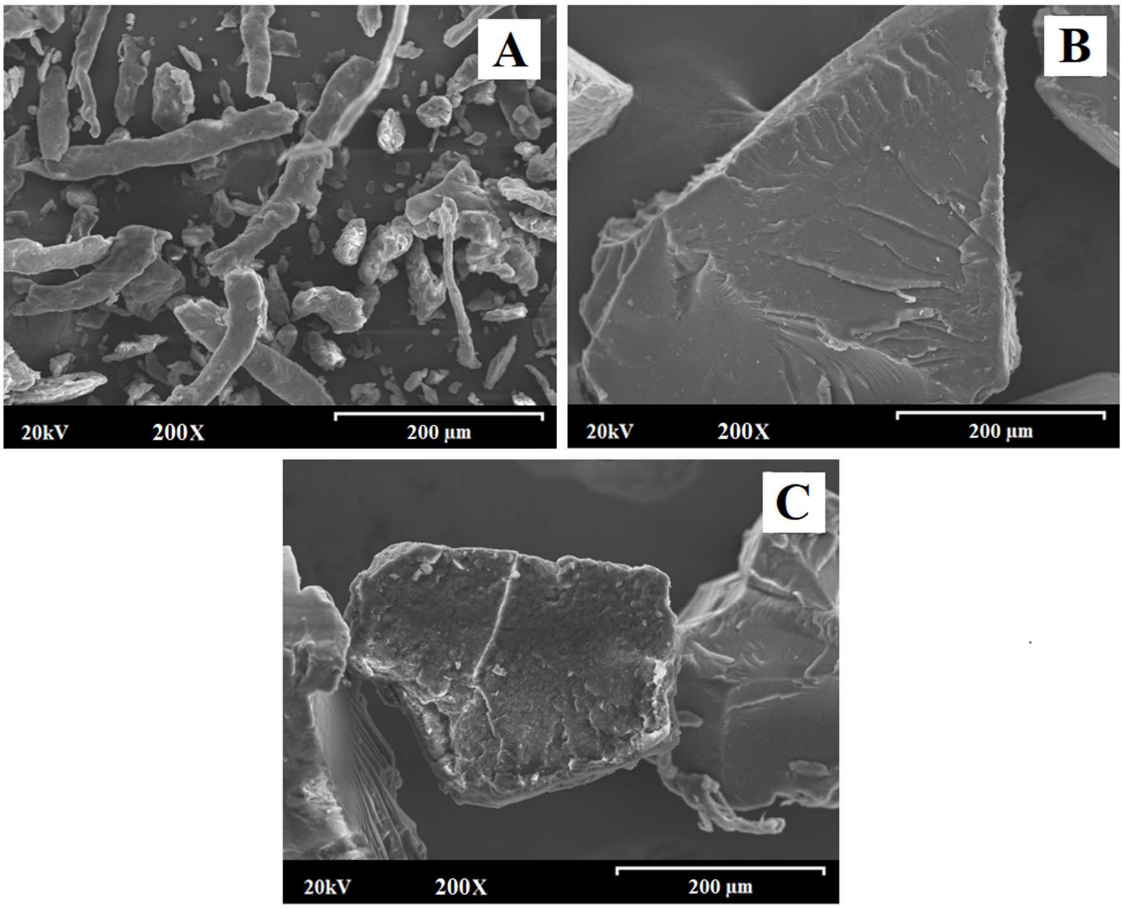

Figure S2. Scanning electron micrographs of polymers at a magnification of 200× and the scale bar is equal to 200 µm: (A) Metolose®; (B) Eudragit®FS and (C) Eudragit®NM.

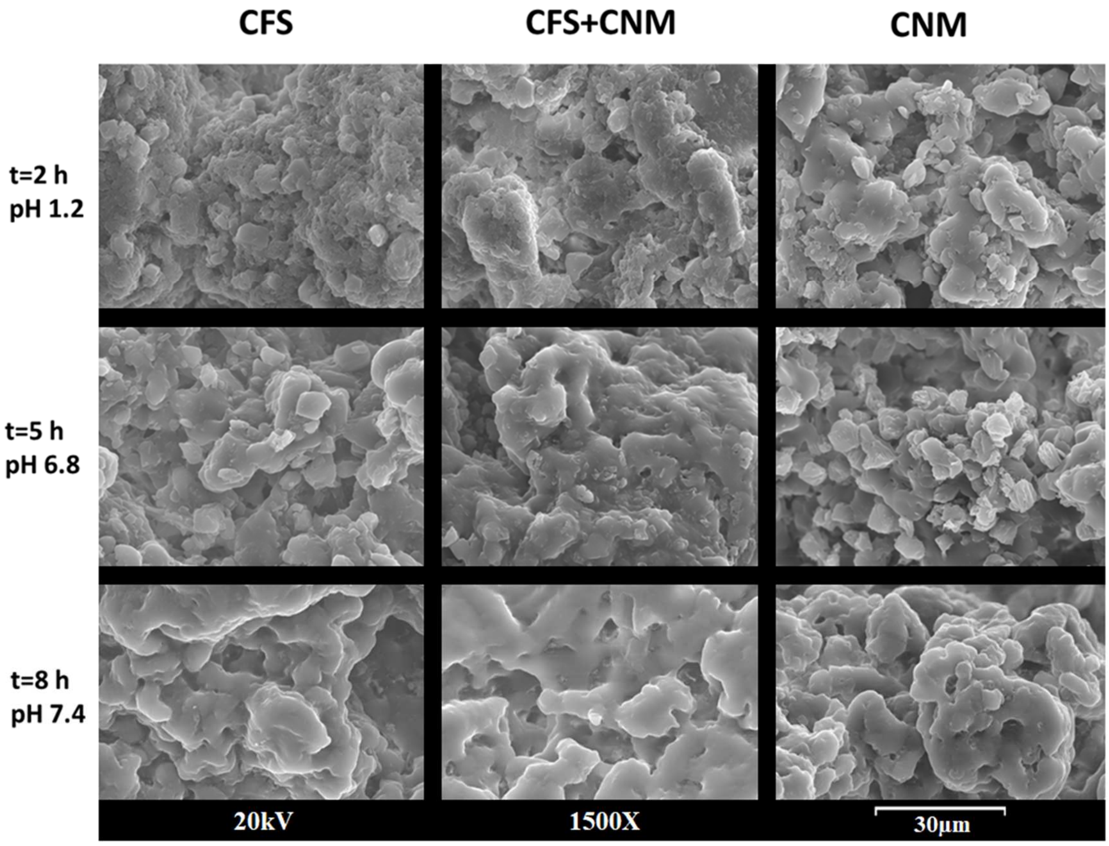

Figure S3. Scanning electron micrographs of CFS, CNM + CFS and CNM multiparticulate systems, after 2 h at pH 1.2; 3h at pH 6.8 and 3 h at pH 7.4. Original magnification is 1500x and the scale bar is equal to 30µm.
